# Pilot Safety Evaluation of a Novel Strain of *Bacteroides ovatus*

**DOI:** 10.3389/fgene.2018.00539

**Published:** 2018-11-06

**Authors:** Huizi Tan, Zhiming Yu, Chen Wang, Qingsong Zhang, Jianxin Zhao, Hao Zhang, Qixiao Zhai, Wei Chen

**Affiliations:** ^1^State Key Laboratory of Food Science and Technology, Jiangnan University, Wuxi, China; ^2^School of Food Science and Technology, Jiangnan University, Wuxi, China; ^3^Wuxi People’s Hospital Affiliated to Nanjing Medical University, Wuxi, China; ^4^National Engineering Research Center for Functional Food, Jiangnan University, Wuxi, China; ^5^International Joint Research Laboratory for Probiotics, Jiangnan University, Wuxi, China; ^6^Beijing Advanced Innovation Center for Food Nutrition and Human Health, Beijing Technology and Business University, Beijing, China

**Keywords:** *Bacteroides ovatus*, safety evaluation, antibiotic resistance, virulence genes, next-generation probiotics

## Abstract

*Bacteroides ovatus* ELH-B2 is considered as a potential next-generation probiotic due to its preventive effects on lipopolysaccharides-associated inflammation and intestinal microbiota disorders in mice. To study safety issues associated with *B. ovatus* ELH-B2, we conducted comprehensive and systematic experiments, including *in vitro* genetic assessments of potential virulence and antimicrobial resistance genes, and an *in vivo* acute toxicity study of both immunocompetent and immunosuppressed mice via cyclophosphamide treatment. The results indicated that this novel strain is non-toxigenic, fragilysin is not expressed, and most of potential virulence genes are correlated with cellular structures such as capsular polysaccharide and polysaccharide utilizations. The antibiotic resistance features are unlikely be transferred to other intestinal microorganisms as no plasmids nor related genomic islands were identified. Side effects were not observed in mice. *B. ovatus* ELH-B2 also alleviated the damages caused by cyclophosphamide injection.

## Introduction

Probiotics, prebiotics, and antibiotics are the most relevant therapies for disorders induced by disturbed microbiota. Traditional probiotics mainly refer to *Lactobacillus* and *Bifidobacterium*, which are normally obtained from traditional fermented foods and are widely accepted as food ingredients or supplements for daily intake, with a prediction of global turnover value of US$46.55 billion by 2020 (O’Toole et al., 2017).

Beneficial strains other than the traditional probiotics have been discovered due to the developments in bacterial culture methodologies and sequencing techniques and have started to be authorized as ingredients in food, particularly from *Bacteroides* which is one of the most abundant genera in the human intestine. For example, *Bacteroides xylanisolvens* DSM23964, which promotes the maturation of natural antibodies against cancers in humans, has recently been permitted to be added to pasteurized milk products under Novel Food Regulation No. 258/97 by the European Commission (Brodmann et al., 2017); *B. uniformis* CECT7771 can improve overweight-induced disorders by reducing the levels of cholesterol and triglyceride (Cano et al., 2012), with no obvious damages identified *in vivo* (Fernandez-Murga and Sanz, 2016).

*B. ovatus* is another dominant species identified as next-generation probiotics either in its original form with sufficient tumor-specific Thomsen–Friedenreich antigen expressed for cancer prevention (Ulsemer et al., 2013), or genetically modified with genes encoding human keratinocyte growth factors (Hamady et al., 2010) or transforming growth factors (Hamady et al., 2011) to facilitate therapies for bowel diseases. However, the beneficial functions of *Bacteroides* are strain-dependent, such as the polysaccharides A (PSA)-producing *B. fragilis* is capable of relieving *Helicobacter hepaticus* associated inflammation and autism spectrum disorders (Mazmanian et al., 2008; Hsiao et al., 2013), but the fragilysin (bft)-carrying *B. fragilis* directly contributes to severe colitis (Yim et al., 2013), emphasizing the necessities for safety evaluations of each potentially beneficial strain.

Previously, we established an efficient method for purifying low-abundant *Bacteroides* species from the human intestine (Tan et al., 2018), and discovered that one of the isolates, *B. ovatus* ELH-B2 displaying promising potentials of modulating lipopolysaccharides (LPS)-induced disorders in cytokine secretions and intestinal microbiota through restoring the balance of regulatory T cells (Tregs) and T helper 17 (Th-17) cells (data unpublished). In this study, a pilot safety assessment of this novel strain was carried out, including explorations of its hemolytic and motile characteristics, antibiotic resistance, genetic virulence factors, and underlying side effects in both normal and immunosuppressed mice.

## Materials and Methods

### Bacterial Strains and Culture Conditions

*Bacteroides ovatus* ELH-B2 was recovered from the in-house preservations at Culture Collections of Food Microbiology (CCFM), Jiangnan University (Wuxi, China). *B. ovatus* JCM5824 was purchased from RIKEN BioResource Center, Japan. *Salmonella enterica* CMCC50335 and *Escherichia coli* CMCC44102 were acquired from the National Center for Medical Culture Collections, China.

*Salmonella enterica* and *E. coli* were anaerobically cultured in brain heart infusion (BHI, Hopebio, China) at 37°C. The *B. ovatus* strains were cultured in BHI supplemented with hemin (Sangon Biotech, China) and vitamin K1 (BHIS) at 37°C in anaerobic chamber for further analysis of bacterial characterizations. The bacteria solutions for *in vivo* tests were prepared with cells at early stationary phase after centrifugation at 6000 rpm for 15 min and re-suspension in phosphate buffer saline supplemented with 20% glycerol, and maintained at -80°C. Cell viability after freezing and thawing was evaluated via colony-forming unit (cfu) enumeration on BHIS agar before use.

### Bacterial Characterizations

*Bacteroides ovatus* type strain JCM5824 was used as control for the bacterial characterization assessments of ELH-B2. Hemolytic capabilities were examined by dropping 5 μl of overnight culture on Brucella agar (Hopebio, China) supplemented with hemin, vitamin K1 and 5% sheep blood (Nanjing SenBeiJia Biological Technology Co., Ltd., China) (Robertson et al., 2006). Motility was tested via standard motility agar assays using BHIS broth supplemented with 0.5% (w/v) agar (soft agar) (Cousin et al., 2015), inoculated with 5 μl of overnight culture and incubated anaerobically for 48 h. *S. enterica* CMCC50335 was adopted as the positive control and *E. coli* CMCC44102 as the negative control. All of the experiments were carried out in three biological replicates.

### Genome Sequencing and Screening of Potential Virulence Factors

**FIGURE 1:**
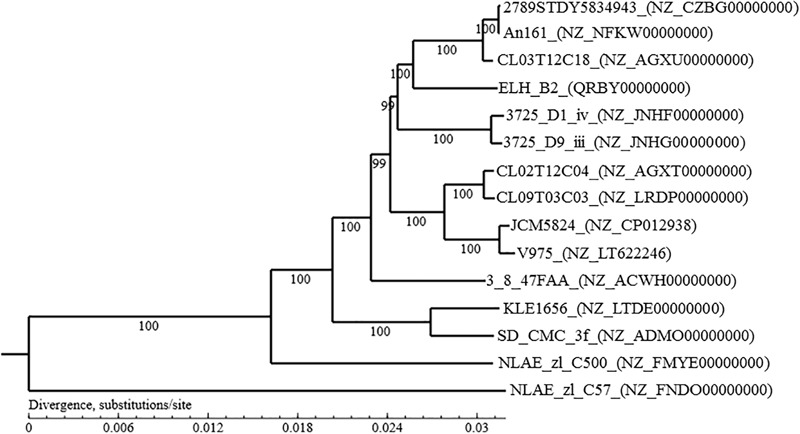
Phylogenetic tree based on the complete genome sequences of *Bacteroides ovatus* ELH-B2 and other *B. ovatus* strains.

The genomic DNA was extracted from *B. ovatus* ELH-B2 culture at early stationary phase and sequenced with Illumina Hiseq system by Majorbio (China). Library of average insert size of 410 bp was generated with low-quality reads filtered. The genome was assembled using SOAPdenovo v2.04^1^ (Li et al., 2008; Li et al., 2010) followed by gap closure and base correction using GapCloser v1.12. A *K*-mer value of 23 was determined according to the accuracy evaluation. Gene annotation was performed by blastp (BLAST 2.2.28+) against Nr, Swiss-prot, string and GO databases. In order to show the relationships between *B. ovatus* ELH-B2 and other *B. ovatus* isolates, whose genome sequences were available from the NCBI database^2^, a neighbor-joining phylogenetic tree (Bottacini et al., 2014) was established by phyML^3^ (Guindon et al., 2009) after alignment of homologous genes identified by graph theory-based Markov clustering algorithm using mafft^4^ (Katoh and Standley, 2013) (Figure 1).

Putative antibiotic resistance genes and virulence genes were identified in the genome of *B. ovatus* ELH-B2 by using a Protein-translated nucleotide Basic Local Search Tool (tblastn) according to the Comprehensive Antibiotic Resistance Database (CARD^5^ (McArthur et al., 2013) and the Virulence Factor Database (VFDB^6^) (Chen et al., 2012) respectively. Positive results were accepted with at least 30% identity and 70% coverage, and e-value less than 0.01 (Salvetti et al., 2016). Genetic islands were also predicted using IslandPath-DIMOB and Islander (Lu and Leong, 2016) for identifying putative virulence factors and possibilities of transportation of antibiotics resistance genes between bacteria. Moreover, *Bacteroides*-specific virulence factors including bft, ompW, upaY, upaZ, wcfR, wcfS, cfiA, cepA, and cfxA, of which the amino acid sequences were acquired from the NCBI database (Table 1B), were screened in *B. ovatus* ELH-B2 using tblastn based on BioEdit v7.2.5 with e-value less than 1e-5. The genome sequence of *B. ovatus* JCM5824 (GenBank accession number NZ_CP012938) was analyzed for comparison.

**Table 1:** **(A)** Identification of potential virulence factors in the genome of *Bacteroides ovatus* ELH-B2 according to VFDB database; **(B)** prediction of putative genomic islands of over 30 kb in the genome of *B. ovatus* ELH-B2; **(C)** comparison of *Bacteroides*-specific virulence genes in *B. ovatus* ELH-B2 and JCM5824.

(A)
**VFDB_ID**	**Name**	**Description**	**Original source**	**Identity**	**Coverage**	***e-*value**

VFG000077	clpP	ATP-dependent Clp protease proteolytic subunit	*Listeria monocytogenes* EGD-e	54%	76%	3.00E-67
VFG000165	pchF	Pyochelin synthetase PchF	*Pseudomonas aeruginosa* PAO1	71%	88%	6.00E-04
VFG000321	rfbD	GDP-D-mannose dehydratase	*Helicobacter pylori* 26695	59%	74%	2.00E-136
VFG000366	ybtQ	Inner membrane ABC-transporter YbtQ	*Yersinia pestis* CO92	56%	72%	3.00E-04
VFG000574	mgtB	Mg2^+^ transport protein	*Salmonella enterica* subsp. *enterica* serovar Typhimurium str. LT2	55%	72%	0.00E+00
VFG000696	bexA	ATP-dependent polysaccharide export protein BexA	*Haemophilus influenzae* str. 1007	49%	78%	1.00E-07
VFG001266	pchI	ABC transporter ATP-binding protein	*Pseudomonas aeruginosa* PAO1	53%	76%	1.00E-05
VFG001269	cyaB	Cyclolysin secretion ATP-binding protein	*Bordetella pertussis* Tohama I	59%	75%	8.00E-04
VFG001301	cap8E	Capsular polysaccharide synthesis enzyme Cap8E	*Staphylococcus aureus* subsp. *aureus* MW2	63%	78%	1.00E-137
VFG001303	cap8G	Capsular polysaccharide synthesis enzyme Cap8G	*Staphylococcus aureus* subsp. *aureus* MW2	57%	72%	1.00E-140
VFG001341	cpsJ	Glycosyl transferase CpsJ(V)	*Streptococcus agalactiae* 2603V/R	58%	78%	6.00E-10
VFG001342	cpsO	Glycosyl transferase CpsO(V)	*Streptococcus agalactiae* 2603V/R	56%	76%	3.00E-14
VFG001374	cps4J	Capsular polysaccharide biosynthesis protein Cps4J	*Streptococcus pneumoniae* TIGR4	72%	85%	1.00E-160
VFG001375	cps4K	Capsular polysaccharide biosynthesis protein Cps4K	*Streptococcus pneumoniae* TIGR4	61%	78%	1.00E-169
VFG001376	cps4L	UDP-*N*-acetylglucosamine 2-epimerase	*Streptococcus pneumoniae* TIGR4	79%	90%	0.00E+00
VFG001855	htpB	Hsp60, 60K heat shock protein HtpB	*Legionella pneumophila* subsp. *pneumophila* str. Philadelphia 1	59%	77%	2.00E-168
VFG001937	Cj1136	Glucosyltransferase	*Campylobacter jejuni* NCTC11168	48%	70%	7.00E-14
VFG001939	Cj1138	Glycosyltransferase	*Campylobacter jejuni* NCTC11168	47%	74%	2.00E-13
VFG001940	wlaN	Beta-1,3 galactosyltransferase	*Campylobacter jejuni* NCTC11168	50%	76%	1.00E-12
VFG001968	Cj1440c	Sugar transferase	*Campylobacter jejuni* NCTC11168	49%	70%	1.00E-11
VFG002181	cpsJ	ABC transporter, ATP-binding protein	*Enterococcus faecalis* V583	58%	76%	1.00E-03
VFG002187	cpsD	Glycosyl transferase, group 2 family protein	*Enterococcus faecalis* V583	36%	71%	3.00E-12
VFG002225	gmd	GDP-mannose 4,6-dehydratase	*Brucella melitensis* bv. 1 str. 16M	69%	81%	2.00E-156
VFG002365	gmd	GDP-mannose 4,6-dehydratase	*Yersinia enterocolitica* 8081	64%	78%	2.00E-151
VFG002368	wbcG	Putative glycosyltransferase	*Yersinia enterocolitica* 8081	50%	78%	2.00E-09
VFG002376	ddhB	CDP-glucose 4,6-dehydratase	*Yersinia enterocolitica* 8081	49%	71%	5.00E-113
VFG002377	ddhA	Glucose-1-phosphate cytidylyltransferase	*Yersinia enterocolitica* 8081	58%	74%	2.00E-94
VFG002440	bprB	Two-component response regulator	*Burkholderia pseudomallei* K96243	50%	72%	6.00E-07
VFG002480	tssH-5/clpV	Clp-type ATPase chaperone protein	*Burkholderia pseudomallei* K96243	56%	74%	2.00E-83
VFG002563	wzt2	ATP-binding ABC transporter capsular polysaccharide export protein	*Burkholderia pseudomallei* K96243	51%	80%	3.00E-06
VFG005773	cylZ	3R-hydroxymyristoyl ACP dehydratase CylZ	*Streptococcus agalactiae* 2603V/R	52%	86%	5.00E-04
VFG011414	kdsA	2-dehydro-3-deoxyphosphooctonate aldolase	*Brucella melitensis* bv. 1 str. 16M	53%	70%	1.00E-65
VFG011430	acpXL	Acyl carrier protein	*Brucella melitensis* bv. 1 str. 16M	64%	80%	2.00E-24
VFG012509	iroC	ATP binding cassette transporter	*Escherichia coli* CFT073	45%	71%	4.00E-05
VFG013354	kfiC	Lipopolysaccharide biosynthesis protein	*Haemophilus influenzae* Rd KW20	47%	83%	2.00E-14
VFG013368	rffG	dTDP-glucose 46-dehydratase	*Haemophilus influenzae* Rd KW20	50%	70%	3.00E-99
VFG013471	lgtA	*N*-acetylglucosamine glycosyltransferase	*Haemophilus influenzae* Rd KW20	48%	80%	2.00E-10
VFG037028	katA	Catalase	*Neisseria meningitidis* MC58	71%	82%	0.00E+00
VFG037386	bauE	Ferric siderophore ABC transporter, ATP-binding protein BauE	*Acinetobacter baumannii* ACICU	42%	70%	8.00E-05
VFG038722	AHA_1389	CobQ/CobB/MinD/ParA family protein	*Aeromonas hydrophila* ATCC7966	51%	71%	5.00E-04
VFG038918	rtxE	RTX toxin transporter, ATPase protein	*Aeromonas hydrophila* ATCC7966	63%	77%	2.00E-05
VFG043373	cheA	Histidine kinase CheA	*Helicobacter pylori* 26695	39%	70%	5.00E-04
VFG043394	cheA	Chemotaxis histidine kinase	*Campylobacter jejuni* NCTC11168	34%	70%	7.00E-04
VFG045340	ricA	Rab2 interacting conserved protein A	*Brucella melitensis* bv. 1 str. 16M	43%	75%	6.00E-07

(B)			
Island no.	Genes in island	Size (kb)	Potential functional genes within island
1	g0188–g0233	37.2	Zeta-toxin (g0214); antitoxin (g0215)
2	g0842–g0866	35.3	Arabinan endo-1,5-alpha-L-arabinosidase (g0844); SusC/RagA family TonB-linked outer membrane protein (g0846); glycosyl hydrolase (g0842, g0851, g0854)
3	g0978–g1164	170	Thiamine biosynthesis protein ApbE (g0984); tonB-linked outer membrane, SusC/RagA family protein (g1005, g1062, g1092); CepA family class A extended-spectrum beta-lactamase (g1025); xylulokinase (g1107)
4	g1544–g1594	34.9	Type IV secretory pathway TrbF components (g1575)
5	g1605–g1720	105.8	Glycosyl transferase 2 family protein (g1616, g1619); two-component sensor histidine kinase (g1646);, TonB-dependent receptor (g1709)
6	g1837–g1864	32.2	Membrane protein (g1855); iron ABC transporter ATP-binding protein (g1864)
7	g2583–g2631	33.3	Redox-active disulfide protein (g2584); thioredoxin (g2585); conjugal transfer protein TraG (g2600, g2610), TraA (g2605), TraH (g2611), TraJ (g2613), TraK (g2614), TraM (g2616), TraN (g2617)
8	g2866–g2999	91.7	Phage Mu F like family protein (g2947); phage tail tape measure protein, TP901 family, core region (g2955); toxin-antitoxin system, antitoxin component, MerR family (g2998)
9	g3114–g3160	41.2	*N*-acetylmuramoyl-L-alanine amidase (g3133)

(C)					
Potential virulence factors	GenBank accession number	Size (aa)	% Similarity in *B. ovatus* ELH-B2	% Similarity in *B. ovatus* JCM5824	Reference
bft	AAF72837.1	63	No hit	No hit	Gutacker et al., 2000
ompW	AAL09385.1	947	95% (904/947)	95% (901/947)	Wei et al., 2001
upaY	AAK68912.1	172	29% (48/163)	30% (48/160)	Coyne et al., 2001
upaZ	AAK68913.1	157	No hit	No hit	
wcfR	AAK68921.1	407	20% (88/407)	25% (101/395)	
wcfS	AAK68922.1	198	48% (68/141)	48% (68/141)	
cfiA	AAK71520	111	No hit	No hit	Gutacker et al., 2002
cfxA	CAP78899.1	306	40% (111/273)	40% (110/273)	Garcia et al., 2008
cepA	AAA21538.1	300	77% (233/299)	77% (233/299)	Rogers et al., 1994

### Minimum Inhibitory Concentration (MIC) of Different Antibiotics

Fourteen antibiotics, corresponding to ampicillin, cefoxitin, ceftriaxone, penicillin G, and vancomycin which suppress cell wall synthesis; chloromycetin, clindamycin, erythromycin, kanamycin, streptomycin, and tetracycline which restrain protein synthesis; ciprofloxacin and metronidazole which inhibit nucleic acid synthesis; and polymyxin B which suppresses cytoplasmic functions, were applied to determine the antibiotic resistance profiles of *B. ovatus* ELH-B2, with the type strain of JCM5824 as comparison. *B. ovatus* overnight culture (100 μl) at a concentration of 10^7^ cfu/ml was treated with serially diluted antibiotics from 0.125 to 1024 μg/ml in sterile 96-well plates. The optical density at 600 nm was checked with a microplate reader (Multiskan GO, Thermo Scientific, United States) after anaerobic cultivation at 37°C for 48 h. The MIC of each antibiotic was determined by the lowest concentration that inhibited 90% of the growth of the tested *B. ovatus* strains (D’Aimmo et al., 2007). All of the experiments were carried out in three biological replicates.

### Animals

Male C57 mice (7 weeks old, spf grade) were purchased from Shanghai Laboratory Animal Center (China) and raised within the IVC rodent caging system at Jiangnan University. The mice were maintained under a 12-h light/dark cycle with temperature and humidity strictly controlled. Treatment was initiated after acclimatization for at least 1 week. The entire experiment was approved by the Animal Ethics Committee of Jiangnan University (JN. No. 20180415c0450730[61]), and protocols for the care and use of experimental animals were based on the European Community guidelines (Directive 2010/63/EU).

### Acute Toxicity to Immunocompetent and Immunosuppressed Mice

Both immunocompetent and immunosuppressed mice were involved in this acute toxicity assessment of *B. ovatus* ELH-B2. The immunocompetent mice, comprising control group (CTRL, 6 mice) and *B. ovatus* ELH-B2 group (BO, 6 mice), were given 150 μl of PBS/glycerol solution or 10^9^ cfu *B. ovatus* ELH-B2 solution by gavage, respectively, every 24 h for 5 days. The other 12 mice were immunosuppressed by intraperitoneal injection with 250 mg/kg of cyclophosphamide (CTX, Sigma-Aldrich, United States), and were allocated to CTX group or CTX + BO group 3 days after followed by daily oral administration of 150 μl of PBS/glycerol solution or 10^9^ cfu *B. ovatus* ELH-B2 solution, respectively, for 5 days (Hirsh et al., 2004; Salva et al., 2014).

The behavior and body weight of each mouse were monitored and recorded throughout the experiments. All of the mice were anesthetized with sodium pentobarbital and sacrificed by cervical dislocation. Liver, spleen and colon tissues and blood samples were collected immediately after sacrifice for further investigations.

### Assays of Hematological and Liver Parameters

Hematological parameters were assessed using automatic hematology analyzer (BC-5000, Mindray, China) and associated buffers with fresh blood samples. The liver parameters were examined using automatic biochemical analyzer (BS-480) and corresponding kits (Mindray, China) with serum obtained by centrifuging the blood samples at 2500 rpm for 10 min. The serum standard (Shanghai Zhicheng Biological Technology Co. Ltd., China) was used for quality control.

### Cytokine Concentrations in Serum

The secretions of tumor necrosis factor alpha (TNF-α), interleukin-6 (IL-6), interleukin-8 (IL-8), and interleukin-10 (IL-10) were determined using mouse Elisa kits purchased from Nanjing SenBeiJia Biological Technology Co., Ltd. (China) with serum samples, according to the manufacturer’s instructions.

### Histological Analysis

Liver, spleen and colon tissues were preserved in 4% paraformaldehyde solution and then embedded in paraffin. The histological analysis was conducted using Hematoxylin-Eosin (H&E) staining as published (Al-Hashmi et al., 2011). Images were recorded using Pannoramic digital slide scanner (Pannoramic MIDI II, 3DHISTECH Ltd., Hungary).

### Statistical Analysis

Significant differences between groups were determined by unpaired Student’s *t*-test using Graphpad Prism v5.0 (Graph Pad Software Inc., United States), with *p*-values of less than 0.05. All of the data were presented as mean ± SD.

## Results

### Microbiological Properties

*Bacteroides ovatus* ELH-B2 grew well at 37°C on the Brucella agar supplemented with laked sheep blood under strict anaerobic conditions. The colonies were round, semi-opaque with smooth edges, and the bacteria were Gram-negative and rod shaped, which matches the description of *B. ovatus* in *Bergey’s Manual* (Krieg et al., 2001). Similar to the type strain *B. ovatus* JCM5824, ELH-B2 was confirmed to be non-motile, but slightly hemolytic.

### Genetic Characteristics and Identification of Potential Virulence Factors

The size of the complete genome of *B. ovatus* ELH-B2 is 1 206 654 732 base pair, including 102 scaffolds and 5909 genes. The GC concent is 41.98%. The genomic information indicates the most similar strain to *B. ovatus* ELH-B2 is *B. ovatus* CL03T12C18 (Figure 1).

According to the blast against VFDB database, 44 virulence factor homologs were identified in *B. ovatus* ELH-B2 (Table 1A), most of which correlate with cellular structures like capsular polysaccharide and polysaccharide utilizations such as glycosyltransferase, and yet have been discovered as the pathogenesis of *B. ovatus*. And there are nine predicted genomic islands of over 30 kb (Table 1B), six of which are metabolism-related. Toxin-antitoxin system-associated genes were discovered two of the potential genomic islands, and one of the components in the Type IV secretion system (T4SS) were also identified.

As for the *Bacteroides*-specific virulence factors (Table 1C), Similar to *B. ovatus* JCM5824, *B. ovatus* ELH-B2 does not contain the diarrhea-associated *B. fragilis* enterotoxin bft. The coding gene of TonB-linked outer membrane protein (ompW), which has been implicated in inflammatory bowel disease (IBD) (Wei et al., 2001), were identified in both *B. ovatus* strains with high similarity. No hits or only low matches were identified in ELH-B2 for the highly conserved open reading frames, upaY and upaZ, and another two genes critical for synthesizing capsular PSA, wcfR and wcfS. And among the three β-lactamase-associated genes, only cepA was found in the two genomic sequences with high similarity.

### Minimum Inhibitory Concentrations of Antibiotics

As shown in Table 2A, the potential antibiotic resistance genes indicated the possibilities of *B. ovatus* ELH-B2 to survive under the treatment of tetracycline, kanamycin, macrolide antibiotics like erythromycin, cationic antibiotics like polymyxin B, and glycopeptide like vancomycin. Accordingly, the MIC experiments verified that *B. ovatus* ELH-B2 was resistant to these antibiotics except tetracycline (Table 2B). It was also clinically susceptible to penicillin, cefoxitin, chloromycetin, and metronidazole with MICs of no more than 32 μg/ml (D’Aimmo et al., 2007). The lowest MIC of ELH-B2 was 4 μg/ml during treatment with metronidazole. Clindamycin and erythromycin were able to inhibit the growth of *B. ovatus* JCM5824 rather than ELH-B2.

**Table 2:** **(A)** Predicted genes associated with antibiotic resistance in the genome of *B. ovatus* ELH-B2; **(B)** minimum inhibitory concentrations for antibiotics against *B. ovatus* ELH-B2 and *B. ovatus* JCM5824 (μg/ml).

(A)
**CARD_ID**	**Name**	**Description**	**Original source**	**Identity**	**Coverage**	***e-*value**

ARO:3000191	tetQ	Tetracycline resistance	*Bacteroides fragilis*	96%	98%	0.00E + 00
ARO:3000197	tet36	Tetracycline resistance	*Bacteroides coprosuis* DSM 18011	60%	79%	0.00E + 00
ARO:3000206	emrK	Major facilitator superfamily (MFS) antibiotic efflux pump	*Escherichia coli*	41%	73%	7.00E-03
ARO:3000250	ErmC	Erythromycin resistance	*Staphylococcus epidermidis*	49%	71%	1.00E-72
ARO:3000375	ErmB	Erythromycin resistance	*Enterococcus faecium*	98%	99%	4.00E-158
ARO:3000499	acrE	Resistance-nodulation-cell division (RND) antibiotic efflux pump	*Escherichia coli* str. K-12 substr. MG1655	46%	73%	5.00E-05
ARO:3000768	abeS	Small multidrug resistance (SMR) antibiotic efflux pump	*Acinetobacter baumannii* AB307-0294	64%	81%	3.00E-26
ARO:3000816	mtrA	Resistance-nodulation-cell division (RND) antibiotic efflux pump	*Mycobacterium tuberculosis* H37Rv	40%	75%	5.00E-07
ARO:3000828	baeR	Resistance-nodulation-cell division (RND) antibiotic efflux pump	*Escherichia coli* str. K-12 substr. W3110	50%	71%	1.00E-08
ARO:3002522	novA	Type III ABC transporter	*Streptomyces niveus*	49%	71%	3.00E-03
ARO:3002626	ANT(6)-Ia	Aminoglycoside nucleotidyltransferase	*Exiguobacterium* sp. S3-2	62%	78%	2.00E-114
ARO:3002627	aadK	Aminoglycoside nucleotidyltransferase	*Bacillus subtilis* subsp. *subtilis* str. 168	58%	76%	1.00E-102
ARO:3002628	aad(6)	Aminoglycoside nucleotidyltransferase	*Streptococcus oralis*	64%	78%	5.00E-107
ARO:3002629	ANT(6)-Ib	Aminoglycoside nucleotidyltransferase	*Campylobacter fetus* subsp. Fetus	73%	87%	2.00E-133
ARO:3002817	carA	ABC transporter involved in macrolide resistance	*Streptomyces thermotolerans*	46%	74%	5.00E-06
ARO:3002825	ErmY	Methyltransferase	*Staphylococcus aureus*	52%	72%	2.00E-70
ARO:3002827	tlrC	Efflux pump	*Streptomyces fradiae*	47%	71%	1.00E-02
ARO:3002830	vgaALC	Efflux protein	*Staphylococcus haemolyticus*	56%	75%	3.00E-04
ARO:3002845	vatH	Acetyl transferase	*Enterococcus faecium*	45%	72%	2.00E-04
ARO:3002871	tet37	Tetracycline resistance	*Uncultured bacterium*	65%	79%	1.00E-31
ARO:3002925	vanRF	Glycopeptide resistance	*Paenibacillus popilliae* ATCC14706	44%	70%	7.00E-15
ARO:3003036	oleB	ABC transporter	*Streptomyces antibioticus*	46%	72%	7.00E-04
ARO:3003048	rosA	Efflux pump for resistance to cationic antimicrobial peptides such as polymyxin B	*Yersinia enterocolitica* (type O:8)	53%	72%	1.00E-96
ARO:3003109	msrE	ABC-efflux pump for resistance to erythromycin	*Escherichia coli*	65%	81%	2.00E-03
ARO:3003548	mdtN	Multidrug resistance efflux pump	*Escherichia coli* str. K-12 substr. W3110	43%	70%	5.00E-04
ARO:3003559	cepA	Beta-lactamase	*Bacteroides fragilis*	78%	89%	3.00E-157
ARO:3003577	ugd	Resistance to cationic antimicrobial peptides	*Escherichia coli* str. K-12 substr. MG1655	58%	72%	7.00E-156
ARO:3003728	vanRI	Glycopeptide resistance gene	*Desulfitobacterium hafniense*	42%	70%	3.00E-17
ARO:3003744	vatF	Streptogramin A acetyl transferase	*Yersinia enterocolitica*	44%	72%	1.00E-08
ARO:3003836	qacH	Small multidrug resistance (SMR) antibiotic efflux pump	*Vibrio cholerae*	53%	71%	1.00E-29
ARO:3003841	kdpE	Adaptive regulator involved in the virulence and intracellular survival of pathogenic bacteria	*Escherichia coli* str. K-12 substr. MG1655	43%	74%	6.00E-06
ARO:3003922	oqxA	RND efflux pump	*Escherichia coli*	56%	72%	2.00E-05
ARO:3003971	erm(44)	Resistance to lincosamide and macrolide antibiotics	*Staphylococcus saprophyticus*	49%	73%	1.00E-67
ARO:3003986	TaeA	Efflux pump	*Paenibacillus* sp. LC231	43%	74%	3.00E-05
ARO:3003987	VatI	Acetyltransferase for resistance to streptogramin A antibiotics	*Paenibacillus* sp. LC231	43%	72%	6.00E-04
ARO:3004038	emrE	Multidrug transporter for resistance to kanamycin	*Pseudomonas aeruginosa* PAO1	50%	73%	2.00E-10
ARO:3004039	emrE	Efflux pump for resistance to erythromycin	*Escherichia coli*	53%	71%	4.00E-30
ARO:3004042	acrA	Resistance-nodulation-cell division (RND) antibiotic efflux pump	*Enterobacter cloacae*	45%	70%	1.00E-03
ARO:3004451	Chloramphenicol acetyltransferase	Chloramphenicol acetyltransferase	*Agrobacterium tumefaciens* str. C58	53%	71%	2.00E-10

(B)		
Antibiotics	*B. ovatus* ELH-B2	*B. ovatus* JCM5824
Ampicillin	MIC ≤ 64	MIC ≤ 128
Penicillin G	MIC ≤ 32	MIC ≤ 32
Vancomycin	MIC ≤ 64	MIC ≤ 64
Cefoxitin	MIC ≤ 16	MIC ≤ 32
Ceftriaxone	MIC ≤ 256	MIC ≤ 128
Clindamycin	MIC ≤ 1024	MIC ≤ 0.25
Chloromycetin	MIC ≤ 16	MIC ≤ 8
Erythromycin	MIC ≤ 1024	MIC ≤ 4
Kanamycin	MIC > 1024	MIC > 1024
Streptomycin	MIC > 1024	MIC > 1024
Tetracycline	MIC ≤ 8	MIC < 0.125
Metronidazole	MIC ≤ 4	MIC ≤ 4
Ciprofloxacin	MIC ≤ 64	MIC ≤ 16
Polymyxin B	MIC ≤ 512	MIC ≤ 128

### *In vivo* Toxicity of *B. ovatus* ELH-B2

**FIGURE 2:**
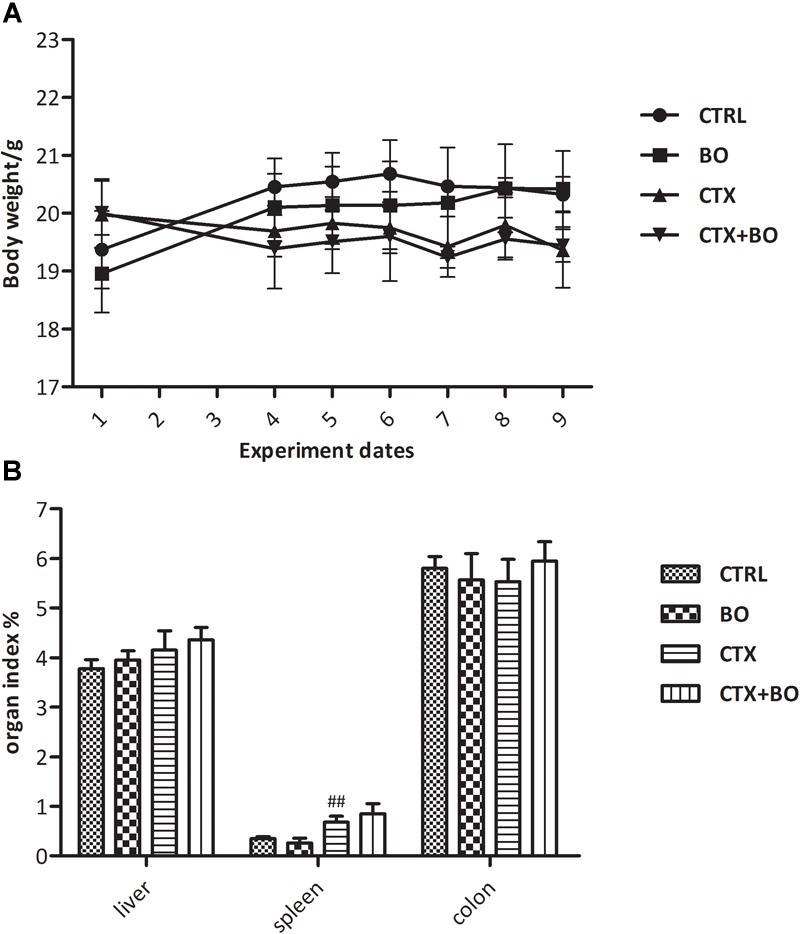
Impact of *B. ovatus* ELH-B2 on **(A)** the body weight and **(B)** the organ indexes of immunocompetent and immunosuppressed mice. The organ indexes of the liver and spleen are expressed as the ratio of the corresponding weight of the organ and body, while the colon index is expressed as the colon length of each animal. Data are displayed as mean ± SD, “##” indicates statistically significant differences between the CTX + BO group and the CTRL group (*p* < 0.01).

All of the animals were alive and healthy at the end of the experiment, and no abnormal behaviors were witnessed. Although the immunosuppressed mice displayed significantly less body weights, *B. ovatus* ELH-B2 treatment did not induce obvious alterations in the body mass of healthy or cyclophosphamide-injected mice (Figure 2A). Concerning the organ index, which refers to the ratio of organ weight to body weight, ELH-B2 had very little effect on liver indexes in immunocompetent mice, and did not enhance the enlarged spleen indexes of the drug-injected mice (Figure 2B). Colon length was also not notably influenced by the administration of ELH-B2.

*B. ovatus* ELH-B2 intervention did not show significant alterations in the hematological (Table 3A) or liver parameters (Table 3B) of normal mice. After CTX injection, the percentages of lymphocytes (*p* < 0.01), hemoglobin concentration (*p* < 0.001), hematocrit value (*p* < 0.01) and platelets enumeration (*p* < 0.05) were markedly dropped, and the percentage of neutrophils were dramatically increased (*p* < 0.01). However, during the treatment with ELH-B2, the hematocrit value of the immunosuppressed mice was recovered (*p* < 0.01) and corpuscular volume was also significantly increased (*p* < 0.01). As for the liver parameters, CTX induced notable upregulation of alanine aminotransferase (*p* < 0.05), and concentration of alkaline phosphatase was dropped to normal level due to the administration of ELH-B2 in CTX-treated mice (*p* < 0.01).

**Table 3:** Profiles of **(A)** hematological values and **(B)** liver parameters in immunocompetent and immunosuppressed mice after 5-day treatments with *B. ovatus* ELH-B2.

(A)
	**CTRL**	**BO**	**CTX**	**CTX + BO**

WBC (10ˆ9/L)	2.47 ± 0.38	2.76 ± 0.64	2.19 ± 0.43	2.21 ± 0.92
Neu (10ˆ9/L)	0.73 ± 0.23	0.40 ± 0.20	0.92 ± 0.38#	1.01 ± 0.65
Lym (10ˆ9/L)	2.56 ± 0.49	2.31 ± 0.48	1.15 ± 0.17##	1.10 ± 0.31
Mon (10ˆ9/L)	0.05 ± 0.02	0.03 ± 0.02	0.08 ± 0.04	0.09 ± 0.05
Eos (10ˆ9/L)	0.02 ± 0.02	0.01 ± 0.01	0.03 ± 0.01	0.04 ± 0.02
Bas (10ˆ9/L)	0.02 ± 0.01	0.01 ± 0.01	0.02 ± 0.01	0.03 ± 0.02
Neu (%)	14.76 ± 5.61	13.90 ± 4.10	40.27 ± 10.93##	39.44 ± 4.86
Lym (%)	83.16 ± 5.67	84.00 ± 4.58	54.17 ± 12.04##	54.95 ± 4.41
Mon (%)	1.30 ± 0.60	1.10 ± 0.58	3.17 ± 1.23#	2.93 ± 0.90
Eos (%)	0.36 ± 0.19	0.57 ± 0.24	1.33 ± 0.40##	1.90 ± 0.37
Bas (%)	0.42 ± 0.13	0.43 ± 0.14	1.07 ± 0.66	1.35 ± 0.23
RBC (10ˆ12/L)	10.44 ± 0.23	10.07 ± 0.46	8.35 ± 0.20	8.88 ± 0.32
HGB (g/L)	171.40 ± 3.14	168.00 ± 3.92	141.33 ± 3.30###	147.50 ± 6.60
HCT (%)	49.78 ± 0.93	48.65 ± 0.70	39.90 ± 0.73###	44.98 ± 2.12**
MCV (fL)	47.72 ± 0.28	48.08 ± 0.38	47.80 ± 0.29	50.17 ± 0.65**
MCH (pg)	16.98 ± 0.17	17.27 ± 0.26	16.93 ± 0.25	16.43 ± 0.27
MCHC (g/L)	356.20 ± 4.71	358.67 ± 5.02	354.00 ± 4.55	356.50 ± 3.59
RDW-CV (%)	12.56 ± 0.24	12.83 ± 0.38	12.63 ± 0.09	13.07 ± 0.59
RDW-SD (fL)	26.88 ± 0.53	27.45 ± 0.73	29.07 ± 0.74	29.77 ± 1.06
PLT (10ˆ9/L)	1227.60 ± 171.16	1059.25 ± 49.44	907.33 ± 109.00#	984.50 ± 160.82
MPV (fL)	5.50 ± 0.24	5.38 ± 0.07	6.00 ± 0.22	5.93 ± 0.22
PDW (%)	15.54 ± 0.12	15.43 ± 0.07	15.77 ± 0.25	15.98 ± 0.17
PCT (%)	0.68 ± 0.10	0.54 ± 0.05	0.55 ± 0.07	0.58 ± 0.09

*WBC, white blood cells; Neu, neutrophils; Lym, lymphocytes; Mon, monocytes; Eos, eosinophils; Bas, basophils; RBC, red blood cells; HGB, hemoglobin concentration; HCT, hematocrit value; MCV, mean corpuscular volume; MCH, mean corpuscular hemoglobin; MCHC, mean corpuscular hemoglobin concentration; RDW, red cell distribution width; CV, coefficient of variation; SD, standard deviation; PLT, platelet; MPV, mean platelet volume; PDW, platelet distribution width; PCT, thrombocytocrit*. *Data are displayed as mean ± SD, “^∗∗^” indicates statistically significant differences between the CTX + BO group and the CTX group (p < 0.01); “#”, “##,” and “###” indicate statistically significant differences between the CTX + BO group and the CTRL group (p < 0.05, p < 0.01 and p < 0.001, respectively)*.

**Table:** 

(B)				
	CTRL	BO	CTX	CTX + BO
Glu (mmol/L)	6.40 ± 0.49	6.59 ± 0.56	7.07 ± 0.96	6.76 ± 0.40
TC (mmol/L)	1.87 ± 0.23	1.90 ± 0.28	1.78 ± 0.28	1.77 ± 0.17
TG (mmol/L)	1.51 ± 0.31	1.58 ± 0.11	1.56 ± 0.45	1.36 ± 0.46
ALT (U/L)	16.30 ± 1.41	17.85 ± 1.57	24.00 ± 3.82#	22.08 ± 1.21
AST (U/L)	175.06 ± 1.42	178.44 ± 13.43	172.50 ± 36.94	180.48 ± 32.45
TBIL (μmol /L)	1.94 ± 0.64	1.96 ± 0.34	2.39 ± 0.43	2.27 ± 0.35
ALP (U/L)	5.25 ± 3.90	5.40 ± 2.24	8.25 ± 3.27	5.00 ± 1.41**
TP (g/L)	40.80 ± 3.17	40.70 ± 4.99	44.10 ± 2.36	44.50 ± 1.47
ALB (g/L)	25.44 ± 2.62	25.05 ± 3.57	27.80 ± 1.81	27.60 ± 1.60
CK (U/L)	2072.58 ± 203.93	2262.52 ± 284.58	2431.00 ± 974.13	2256.23 ± 676.64
LDH (U/L)	423.83 ± 228.77	465.80 ± 69.24	511.00 ± 63.94	516.30 ± 62.95

*Glu, glucose; TC, total cholesterol; TG, triglyceride; ALT, alanine aminotransferase; AST, aspartate aminotransferase; TBIL, total bilirubin; ALP, alkaline phosphatase; TP, total protein; ALB, albumin; CK, creatine kinase; LDH, lactic dehydrogenase.*

**FIGURE 3:**
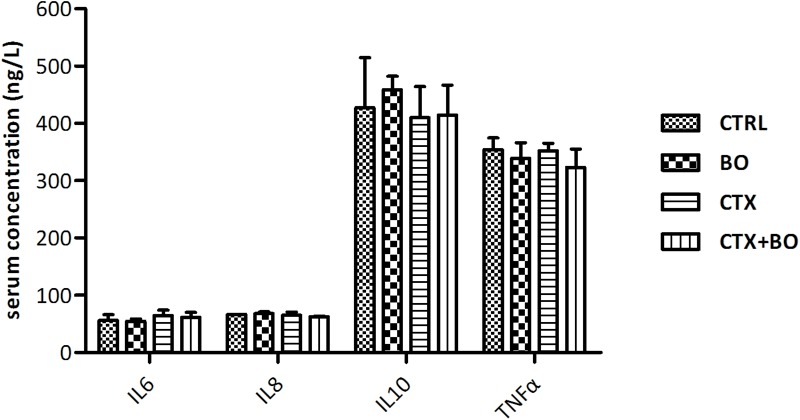
Impact of *B. ovatus* ELH-B2 on the cytokine productions of immunocompetent and immunosuppressed mice. Data are displayed as mean ± SD.

**FIGURE 4:**
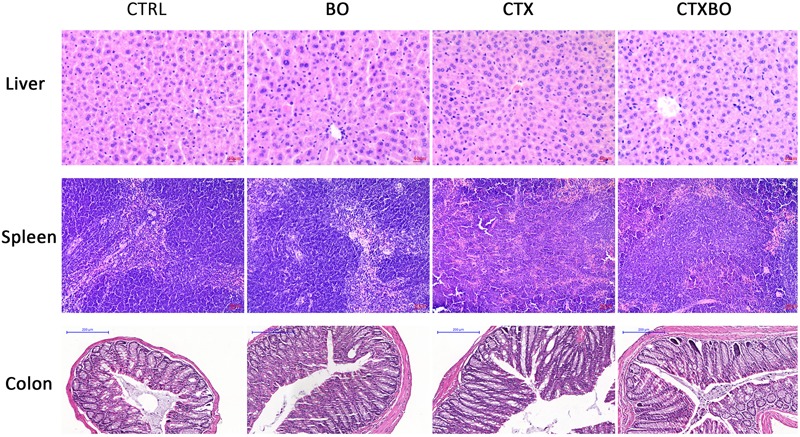
Impact of *B. ovatus* ELH-B2 on the tissue histology of immunocompetent and immunosuppressed mice.

No obvious modifications in cytokine productions were observed after the treatment of *B. ovatus* ELH-B2 in both healthy and immunosuppressed mice (Figure 3). Treatment with *B. ovatus* ELH-B2 did not lead to any histopathological damage in the liver, spleen or colon of the healthy mice (Figure 4). However, the CTX-treated mice suffered from hypertrophy of spleen, the histological structure of which was severely damaged with obvious fibrosis and hemorrhage. The red and white pulps could not be well-defined and splenocytes were irregularly aligned (Figure 4).

## Discussion

Evidences of indigenous and genetically modified intestinal commensals which obtain underlying efficacy in modulating immune and metabolic disorders, extend the range of probiotics, and are termed “next-generation probiotics” or “live biotherapeutic products.” The United States Food and Drug Administration (FDA) provide a definition for live biotherapeutic products as “a biological product that contains live organisms, such as bacteria, and is applicable to the prevention, treatment or cure of a disease or condition of human beings, but is not a vaccine,” which is also suitable for next-generation probiotics (O’Toole et al., 2017). In the meantime, the FDA drafted guidance that next-generation probiotics should be authorized as food ingredients when first entering the market. However, specific guidelines for applications of these promising microorganisms are yet to be developed. Therefore, a safety evaluation of *B. ovatus* ELH-B2 was carried out according to the regulations of the FAO/WHO for development of probiotics, in which explained the importance of complete bacterial characterizations such as original source, culture history, phenotype and genotype, antibiotic resistance, and manufacturing methods and three-step clinical trials, including safety assessment and functional characterization; double blind, randomized, placebo-controlled human studies; and efficiency comparisons with standard treatments, and published toxicity analyses of *Lactobacillus* spp. (Yakabe et al., 2009; Jia et al., 2011), *B. xylanisolvens* DSM23964 (Ulsemer et al., 2012a,b), *B. uniformis* CECT7771 (Fernandez-Murga and Sanz, 2016), and *B. fragilis* ZY312 (Wang et al., 2017), along with previous results which revealed that ELH-B2 had little effect on the production of secretory immunoglobulin A (sIgA) and chemokine (C-X-C motif) ligand 2 (CXCL2) or the balance of Treg and Th-17 cells, and even upregulated the diversity of intestinal microbiota (unpublished data).

Morphological analysis showed that *B. ovatus* ELH-B2 cells were non-motile, which excluded the pathogenic factor of flagella for inducing inflammation via activating the NF-κB pathway through Toll-like receptor 5 and secreting IL-8 (Neville et al., 2012), and facilitating nutrient acquisition, niche colonization (Lane et al., 2007; Neville et al., 2012), and biofilm formation (Houry et al., 2010). The translucent circles around ELH-B2 colonies on blood agar plates indicated the possible existence of hemolysin, which is a pore-forming toxin with cytolytic functions on various types of cells, such as keratinocytes, epithelial cells, and lymphocytes (Kennedy et al., 2010; Wilke and Wardenburg, 2010).

The virulence factors discovered via the Virulence Factor Database include genes facilitating protein secretion, carbohydrates degradation and maintaining cellular structures. These elements could be probiosis-related and contribute to bacterial adhesion and colonization, rather than pathogenicity (Wassenaar et al., 2015). The majority of the predict islands were discovered to be metabolism-related. Although TrbF of T4SS were identified, the rest preserved genes were absent. Thus *B. ovatus* ELH-B2 are not capable of producing the entire secretion systems for any possibilities of transfering virulence protein and antibiotic resistance genes (Aguilar et al., 2010). Moreover, toxin-antitoxin system are widely existed in natural bacteria strains for better adaptive ability to the environment resulted from evolution (Buts et al., 2005), the virulence characteristics of which requires further analysis.

*Bacteroides* species are, to some extent, considered to be opportunistic pathogens as some of them are carriers of virulence factors, such as the enterotoxigenic *B. fragilis* with bft (Sears, 2009) and *B. caccae* with ompW (Wei et al., 2001). The metalloprotease bft was not identified in ELH-B2, but ompW was found to be 95% identical. The protein encoded by ompW in *B. caccae* was discovered by pANCA monoclonal antibody in IBD patients and is closely associated with a pathogenic factor of *Porphyromonas gingivalis* that contributes to tissue damage. However, this TonB-linked structure of ompW is also conserved in the starch-utilization system of *Bacteroides* species that helps to break down resistant carbohydrates in the host. Hence, the underlying role of the ompW-like structure in ELH-B2 requires further investigation. Meanwhile, the blast results of the four genes essential for constructing PSA indicated the absence of the capsular polysaccharide, which suggests that ELH-B2 does not possess the PSA-associated anti-inflammatory character (Mazmanian et al., 2008), but also avoids the consequently potential abscess formation (Cohen-Poradosu et al., 2011).

Overall, the putative antibiotic resistance gene list based on the Comprehensive Antibiotic Resistance Database are almost in correspondence to the antibiotic resistance profiles of *B. ovatus* ELH-B2 acquired from MIC experiments. *B. ovatus* ELH-B2 was found to be less susceptible to antibiotics compared with the type strain. The three genes encoding β-lactamase, corresponding to cfxA for class A cephalosporinase, cfiA for class B metallo-β-lactamase and cepA for endogenous cephalosporinase (Garcia et al., 2008), were not or were only partially aligned in the genome of ELH-B2, which was consistent with the result that the novel strain was clinically resistant to penicillin G and cefoxitin. It is notable that neither plasmid nor antibiotic resistance-related genomic islands were found in the genome, indicating low chances of transferring the characteristics of antibiotic resistance to other intestinal commensals.

In general, the results demonstrated that *B. ovatus* ELH-B2 showed no oral pathogenicity in healthy animals with a daily dose of 10^9^ cfu live cells, which is the appropriate concentration for commercial application that guarantees both the viability and integrity of bacterial cells after freeze-drying and restoration procedures (Miyamoto-Shinohara et al., 2000). Based on a mean body weight of 20 g for the mice, 3.5 × 10^12^ cfu of ELH-B2 cells are predicted to be safe for a 70 kg healthy human adult.

In the meantime, confidence in authorizing treatments for industrial and clinical applications would be enhanced once no side effects of the bacteria have been confirmed in immunodeficient animals (FAO/WHO, 2002). In this study, immunosuppressed condition was established via CTX injection, which is one of the most commonly used as a chemotherapeutic treatment for cancers and as an immunosuppressive agent before myeloablative therapies (Ehrke, 2003). Accordingly, the cytotoxicity was reflected in liver which is the first target organ engaging all toxic drugs (Singh et al., 2018), and spleen which is responsible for the immune status by controlling the proliferation of T cells, B cells and lymphocytes (Gong et al., 2015). CTX treatment led to distinctly upregulated liver parameter of alanine aminotransferase, injuries in spleen and disturbed hematological values.

Nevertheless, *B. ovatus* ELH-B2 did not accelerate the toxicities of CTX. Besides, the restoration of alkaline phosphatase revealed a recovery effect on the liver and pancreatic functions. Alkaline phosphatase contributes to the dephosphorylation of LPS from the gut by circulation (Moreira et al., 2012), and thereby the downregulation of which emphasized the capability of the *Bacteroides* to help reduce the threats of endotoxemia in the mice. This result corresponds to the previous characterization study of this novel strain. A reduction in the secretion of TNF-α was observed with *B. ovatus* ELH-B2 treatment, demonstrating its potential anti-inflammatory function, although in a non-significant way.

In summary, *B. ovatus* ELH-B2, a novel strain which was confirmed to be capable of attenuating LPS-induced inflammation *in vivo*, did not raise severe safety issues in either immunocompetent or immunosuppressed mice, and even partially relieved the side effects associated with the chemotherapeutic drug. Further assessments of viable doses and extreme conditions, such as intraperitoneal injection of bacteria, should be considered (Ulsemer et al., 2012b).

## Author Contributions

HT and ZY carried out the experiment and drafted the manuscript. CW and QinZ participated in the analysis of the data. JZ and HZ provided the essential reagents and materials. QixZ conceived of the study and managed the project design. WC helped to revise the manuscript. All authors read and approved the final manuscript.

## Conflict of Interest Statement

The authors declare that the research was conducted in the absence of any commercial or financial relationships that could be construed as a potential conflict of interest.

## Footnotes

1 http://soap.genomics.org.cn/

2 https://www.ncbi.nlm.nih.gov/

3 http://www.atgc-montpellier.fr/phyml/

4 https://mafft.cbrc.jp/alignment/software/

5 http://arpcard.mcmaster.ca/

6 http://www.mgc.ac.cn/VFs/main.htm
